# Protocol for a systematic review and meta-analysis of the diagnostic accuracy of artificial intelligence for grading of ophthalmology imaging modalities

**DOI:** 10.1186/s41512-022-00127-9

**Published:** 2022-07-14

**Authors:** Jessica Cao, Brittany Chang-Kit, Glen Katsnelson, Parsa Merhraban Far, Elizabeth Uleryk, Adeteju Ogunbameru, Rafael N. Miranda, Tina Felfeli

**Affiliations:** 1 Department of Ophthalmology and Vision Sciences, University of Toronto, Toronto, Ontario Canada; 2 Faculty of Medicine, University of Toronto, Toronto, Ontario Canada; 3 Faculty of Medicine, Queens University, Kingston, Ontario Canada; 4 E/M Uleryk Consulting, Mississauga, Ontario Canada; 5 Institute of Health Policy, Management and Evaluation, Dalla Lana School of Public Health, University of Toronto, Toronto, Ontario Canada; 6 THETA Collaborative, Toronto General Hospital, University Health Network, Eaton Building, 10th Floor, 200 Elizabeth Street, Toronto, Ontario ON M5G Canada

**Keywords:** Ophthalmology, Artificial intelligence, Diagnostic accuracy, Image grading, Meta-analysis

## Abstract

**Background:**

With the rise of artificial intelligence (AI) in ophthalmology, the need to define its diagnostic accuracy is increasingly important. The review aims to elucidate the diagnostic accuracy of AI algorithms in screening for all ophthalmic conditions in patient care settings that involve digital imaging modalities, using the reference standard of human graders.

**Methods:**

This is a systematic review and meta-analysis. A literature search will be conducted on Ovid MEDLINE, Ovid EMBASE, and Wiley Cochrane CENTRAL from January 1, 2000, to December 20, 2021. Studies will be selected via screening the titles and abstracts, followed by full-text screening. Articles that compare the results of AI-graded ophthalmic images with results from human graders as a reference standard will be included; articles that do not will be excluded. The systematic review software DistillerSR will be used to automate part of the screening process as an adjunct to human reviewers. After the full-text screening, data will be extracted from each study via the categories of study characteristics, patient information, AI methods, intervention, and outcomes. Risk of bias will be scored using Quality Assessment of Diagnostic Accuracy Studies (QUADAS-2) by two trained independent reviewers. Disagreements at any step will be addressed by a third adjudicator. The study results will include summary receiver operating characteristic (sROC) curve plots as well as pooled sensitivity and specificity of artificial intelligence for detection of any ophthalmic conditions based on imaging modalities compared to the reference standard. Statistics will be calculated in the R statistical software.

**Discussion:**

This study will provide novel insights into the diagnostic accuracy of AI in new domains of ophthalmology that have not been previously studied. The protocol also outlines the use of an AI-based software to assist in article screening, which may serve as a reference for improving the efficiency and accuracy of future large systematic reviews.

**Trial registration:**

PROSPERO, CRD42021274441

**Supplementary Information:**

The online version contains supplementary material available at 10.1186/s41512-022-00127-9.

## Background

Imaging is an important diagnostic and prognostic tool in ophthalmic patient care. With the ever-increasing use of diagnostic imaging in technology, there is a growing need for accurate and efficient grading of ophthalmic images for informing patient care [1]. Much research has been done in recent years into artificial intelligence (AI) systems that can analyze ophthalmic images and provide an accurate screening result [2, 3]. With the increasing number of patients screened through teleophthalmology, there is a growing demand for experienced human graders such as subspecialty ophthalmologists [4–6]. Deep learning has already shown promise in ophthalmic image recognition capabilities [7]. Previous systematic reviews have shown that AI has a sensitivity of 80–100% and a specificity of 84–100% in diagnosing diabetic retinopathy, a condition involving the posterior segment of the eye, from fundus photographs [8, 9]. The use of AI in anterior segment diseases has been also explored more recently in a multicenter study, which suggested a sensitivity and specificity of 89.7% and 86.4%, respectively, in the diagnosis of pediatric cataracts [10]. Advancements in automated image analysis are valuable to eye disease screening programs, particularly those in day-to-day disease risk prediction and virtual care such as teleophthalmology, which aim to reduce barriers to care, particularly in underserved populations [11]. Machine learning models such as convolutional neural networks are used in medical image analysis to automate recognition and diagnosis [12].

Published systematic reviews on the topic of diagnostic accuracy of AI for grading ophthalmic images have had a narrow scope limited to a few ophthalmic conditions such as diabetic retinopathy, age-related macular degeneration, glaucoma, and retinopathy of prematurity [7, 13]. Previous studies have recognized the potential of AI for use in other applications in ophthalmology, but this information has yet to be synthesized and reviewed critically [9]. More information is needed on the specific AI tools available, as well as their reliability in providing accurate diagnoses in all clinical contexts of ophthalmology including assistance in clinical decision-making [14].

Given the rise of AI in medicine and ophthalmology, defining its accuracy, and reliability, will guide future research in this area and enhance its real-life adaption. This review aims to elucidate the diagnostic accuracy of artificial intelligence in screening for all ophthalmic conditions in patient care settings that involve digital imaging modalities, using the reference standard of human graders.

## Methods

### Study design

This is a systematic review and meta-analysis. This protocol is registered in PROSPERO (CRD42021274441).

### Study objectives

This project aims to determine the diagnostic accuracy of AI in ophthalmology clinical settings, with results stratified by and presented for each ophthalmic condition. Where sufficient information is available, patients will also be grouped by age, either pediatric (under 18 years of age) or adult (18 years or older). Some ophthalmic conditions, such as retinopathy of prematurity, occur exclusively in the pediatric population, whereas others such as age-related macular degeneration occur most commonly in senior adults. Studies with a mix of patient ages will be characterized based on the proportion of adult and pediatric patients. Both these examples provide the potential for AI-assisted screening through automated grading of various diagnostic imaging modalities.

The present study will further subgroup ophthalmic conditions by their anatomic location. Anterior segment conditions include cataract, keratoconus, and dry eye disease. Common forms of imaging include anterior segment optical coherence tomography (AS-OCT), keratometry, and slit lamp photography. Posterior segment conditions such as diabetic retinopathy, age-related macular degeneration, and open-angle glaucoma can be visualized via imaging modalities such as OCT of the macular and optic nerve, fundus photography, and visual field testing.

Additional subgroups for studies will be based on the setting of clinic or remote via teleophthalmology. This will allow the authors to discern whether the patient setting is related to the diagnostic accuracy of AI.

For all analyses, human graders will serve as the reference standard and will assess the diagnostic accuracy of the AI screening results relative to images graded by humans. Human graders were set as our reference standard as human grading is the predominant and best method thus far in providing a diagnosis. As diagnoses can differ between the eyes for each individual, this study will use the eye as the unit of analysis.

The results will be reported according to the Preferred Reporting Items for Systematic Reviews and Meta-Analyses for Diagnostic Test Accuracy (PRISMA-DTA) guidelines [15].

### Search strategy

We will undertake a literature search of relevant articles using a comprehensive search strategy developed in consultation with experienced librarians. The search will be conducted on Ovid MEDLINE, Ovid EMBASE, and Wiley Cochrane CENTRAL for articles from January 1, 2000, to December 20, 2021. The timeline of 2000 as the initial search start date was chosen to reflect the recency of AI development and application, including one of the first studies using AI in ophthalmology, which was published in 2004 [16]. The search will include a group of terms related to artificial intelligence and ophthalmology. Subject headings as well as key terms will be included. The search was first developed on Ovid MEDLINE, then translated to Ovid EMBASE and Wiley Cochrane CENTRAL. The search will not be restricted based on language or patient population. Additional file 1 includes the complete search strategy for all three databases.

### Study selection

#### Inclusion and exclusion criteria

Peer-reviewed scientific articles found in the chosen databases that compare the results of AI-graded ophthalmic images with results from human graders will be included. The scope of imaging for ophthalmic conditions will include, but are not limited to, keratoconus, cataract, angle-closure glaucoma, dry eye disease, posterior capsule opacification, diabetic retinopathy, age-related macular degeneration, retinopathy of prematurity, open-angle glaucoma, epiretinal membrane, and macular hole. Patients of any age or comorbidity status will be included. Studies that report the outcomes of interest including false positive, false negative, true positive, true negative, or sensitivity and specificity will be included.

Review papers, case reports, conference abstracts, guidelines, editorials, commentaries, and opinion pieces will be excluded. Papers not in English will be excluded. Articles that do not compare the performance of AI versus human graders will be excluded.

#### Software used

Due to the large number of anticipated studies from the search, the systematic review software DistillerSR (Evidence Partners) was chosen to assist with deduplication of citations and screening of articles [17]. DistillerSR uses machine learning to automate part of the screening process as an adjunct to human graders [18]. After providing the software with a training set where reviewers manually provide the screening result, the DistillerSR software will recognize the patterns and keywords used for screening that can be applied to the remainder of the articles. A relevance threshold level can be set to control the strictness of screening, and manual checks are available at various steps to ensure the desired screening result. Using this software will allow a much broader scope to be accomplished than previous systematic reviews on the topic. A user study conducted in 2019 evaluating the accuracy of DistillerSR AI software in semi-automated screening demonstrated a combined sensitivity of 78% (95% CI, 66 to 90%) and a combined specificity of 95% (95% CI, 92 to 97%) of the AI software compared to human reviewers [19]. This was comparable to a single reviewer’s sensitivity performance and exceeded the sensitivity of using the DistillerSR software alone. The results of the user study thus informed the decision to semi-automate this study’s screening.

All statistical analysis for the meta-analysis will be completed with R.

#### Screening of studies

Retrieved studies from the searched databases will be imported into the systematic review software DistillerSR and deduplicated. Studies will be selected via a two-stage screening process, first by screening titles and abstracts, followed by full-text screening. The screening process will be supplemented with DistillerSR using a stepwise approach. After undergoing training on inclusion and exclusion criteria, two independent reviewers will screen the papers until a minimum of 10 relevant articles are selected for inclusion and a total of 1500 articles screened are reached. This will serve as the training set for the automated DistillerSR screening software. For the next set of 500 articles or more, one reviewer will screen the titles and abstracts, and DistillerSR will be used as the second reviewer. These thresholds were chosen as a conservative approach to screening based on manufacturer recommendations for optimal performance of the software. A relevance threshold will be set at 0.1 (most conservative threshold chosen to ensure high sensitivity for inclusion of studies and prevent exclusion of any relevant articles). If an acceptable level of agreement (> 90%) between the reviewer and DistillerSR is achieved, the remaining set of articles will be graded by DistillerSR alone [20]. In this case, a quality check of a random selection of 10% of articles screened by DistillerSR alone will be done by a reviewer to ensure no relevant studies are excluded [4]. In case an acceptable level of agreement is not achieved (< 90%), the algorithm will be re-run with the inclusion of newly screened articles to increase the training set size. Below the relevance threshold of 0.1, we will use DistillerSR only for screening. If again the level of agreement is < 90%, then one reviewer will screen the papers with DistillerSR serving as the second screener. In all steps, any disagreements will be reviewed by a third senior adjudicator.

### Data extraction

After full-text screening, data will be extracted from each study via the categories of study characteristics, patient information, AI methods, and outcomes (e.g., sensitivity and specificity) (BCK and GK). The full list of data categories to be extracted is presented in Table 1.

**Table 1:** Data to be extracted from each study

Data category	Collected data
Study characteristics	- Primary author
	- Publication year
	- Recruitment period/study duration
	- Country
	- Study purpose
	- Study type (e.g., RCT, prospective cohort study)
	- Sample size
	- Clinical setting (academic/community)
	- Reference standard description (e.g., human graders—retina specialists)
	- Ophthalmic condition screened for
	- Funding sources
	- Follow-up period
Patient information	- Patient sociodemographic data (including age (mean/median and categorization of pediatric and adult), sex, comorbidities, eye conditions, race/ethnicity, income status, education)
	- Inclusion and exclusion criteria
AI methods	- Imaging modalities used for screening (e.g., fundus photographs, ocular coherence tomography)
	- Automated algorithms or tools used (boosted tree, random forest, etc.)
	- Role of AI in screening
	- Number of human graders
	- Number of ungradable images
	- Identified pathologies (types and proportions)
Intervention outcomes	- Sensitivity/specificity
	- Positive predictive value
	- Negative predictive value
	- % correct as analyzed by artificial intelligence
	- Diagnostic accuracy (if stated)

### Assessment of study quality

Quality Assessment of Diagnostic Accuracy Studies (QUADAS-2) will be used by two independent reviewers to assess the quality of included studies based on the 4 domains of index test, reference test, patient selection, and flow/timing [21]. Multiple signaling questions for each domain guide the bias review. The risk of bias is graded as high, low, or unclear. A grading of unclear is given only if there is insufficient information to make a decision. If at least one signaling question is answered as “no,” there is a potential for bias, and reviewers will independently judge the risk for bias. Unclear grading results when there is insufficient data for a judgment to be made.

In cases where studies exclude patients from the comparative analysis, we established a low risk-of-bias cutoff at 10% of ophthalmic images that were deemed ungradable by the human graders. This cutoff was informed by a selection of review papers, which labeled a 5–10% ungradable rate as low [22, 23].

Any disagreements in grading will be reviewed by a third adjudicator. A summary and graphic representation of the QUADAS-2 gradings for all studies will be presented in the final review. A sensitivity analysis will be conducted by removing studies with a high risk of bias.

### Missing data

Where there is missing data, we will make attempts to contact the corresponding author of the studies through the email listed in the publication. A total of three attempts will be made. If no response is received, the authors will make the best attempt to perform the analysis based on available data and code any data not available as missing. The missing data will be noted as a limitation in the discussion section of the manuscript.

### Data synthesis

For each study, screening outcomes via artificial intelligence will be entered in a two-by-two table (true positive, false positive, true negative, false negative). The data of the two-by-two tables will be used to calculate the sensitivity and specificity for each study (Table 2). We will present individual study results graphically by plotting the estimates of sensitivity and specificity in both forest plots and on the summary receiver operating characteristic (sROC) curve plots. The individual area under curve (AUC) measures will be combined to calculate the area under the sROC [24]. Negative predictive value (NPV) and positive predictive value (PPV) will not be used in the meta-analyses, given their dependence on the underlying prevalence of disease in artificially constructed study populations. NPV and PPV will be reported descriptively where available, and limitations to data interpretation will be highlighted for the readers.

**Table 2:** Sample two-by-two contingency table used for analysis

		Reference (result by human graders)	
		Positive	Negative
**Test (AI result)**	**Positive**	True positive	False positive
	**Negative**	False negative	True negative

We will also conduct a subgroup analysis on the diagnostic accuracy of artificial intelligence when used specifically in teleophthalmology programs.

Separate analyses will be conducted for the most common imaging modalities (e.g., ocular coherence tomography, color fundus images, visual fields), to allow comparison between modalities when sufficient data is available.

In cases where multiple AI techniques are applied in the same study, the technique with best reported sensitivity and specificity will be selected for analysis. Comparison of multiple AI techniques is commonly done in validation studies, and the final outcomes are often reported based on the most optimally performing AI technique. If sufficient data is available, additionally, we will stratify all used AI techniques and report the subsets accordingly.

Our unit of analysis is the eye, given that each eye may have a separate diagnosis and therefore affect accuracy in different ways. Some studies may only report results per patient instead of per eye. As such, a sensitivity analysis will be conducted with the unit of analysis as each patient to ensure consistency of results.

Pooled sensitivity and specificity of artificial intelligence for detection of any ophthalmic conditions based on imaging modalities compared to the reference standard (i.e., human graders) will be reported. The findings will be stratified by ophthalmic condition (anterior vs posterior segment disease entities; when sufficient data is available), as well as demographics (pediatric vs adults ≥ 18 years old). Pooled estimates of the sensitivity and specificity will be obtained with random effect models, using the DerSimonian-Laird method to incorporate variation among studies [25]. In addition to the random effects model, we will build a fixed effects model as a sensitivity analysis. This will be done to compare the robustness of the random effects model against the fixed effects model, as the random effects model is a more conservative approach.

We will investigate the heterogeneity firstly through visual examination of forest plots of sensitivities and specificities, as well as the sROC plot of the raw data. Last, we will use Cochran’s *Q* test to evaluate homogeneity. We will also use the statistic *I*^2^ of Higgins to quantify the amount of heterogeneity. The scale of *I*^2^ has a range of 0 to 100%, and values of 25%, 50%, and 75% are considered low, moderate, and high heterogeneity, respectively. All statistical analyses will be completed by qualified biostatisticians (PNM, AO, RNM).

## Discussion

This systematic review will aim to identify the diagnostic accuracy of AI in image recognition for ophthalmic diseases. This will be the first review to our knowledge with a broad scope with no restriction on the type of ophthalmic condition, which will allow a thorough assessment of AI accuracy and reliability. The study findings can help clinicians to ascertain as to whether certain types of image analysis and screening can be allocated to AI systems, thus reducing healthcare resource utilization.

AI and automated image grading are most commonly used in some posterior segment pathologies such as diabetic retinopathy and age-related macular degeneration. Accordingly, we expect to find many studies on these conditions and have enough data to calculate a pooled sensitivity and specificity via meta-analysis. We anticipate that we will have between 2 and 6 conditions available with sufficient data for meta-analyses. A meta-analysis for a given condition will not be conducted if there are 2 or fewer studies. However, the use of AI has more recently been explored in other ophthalmic conditions. As such, data may be insufficient or show that current AI systems still need refinement before making reliable diagnoses. Similarly, the body of research on adult ophthalmic conditions is much wider compared to pediatric conditions. As such, we anticipate more AI systems as well as better diagnostic accuracy in adult ophthalmic pathologies due to the higher availability of training data.

We also anticipate the differences to be noted in the diagnostic accuracy of AI based on the setting of use. Clinic settings have the additional benefit of the patient being present for a full history and clinical exam. Clinicians can rely on ancillary information to make a diagnosis, and thus, the grading result of specific ophthalmic imaging may be less applicable. However, in the context of remote screening via teleophthalmology, human graders can only rely on limited clinical information in addition to imaging. Thus, AI may be able to play a more important role in teleophthalmology programs.

There are some limitations to this study. Firstly, given that the quality and quantity of data in less common conditions are unknown, we may not be able to conduct an accurate meta-analysis and provide pooled sensitivity and specificity values for some diseases. Other potential limitations of the study include the reliability of human graders which serve as the reference standard. In our analysis, the assumption is made that human graders have 100% sensitivity and specificity. There may be certain scenarios where human graders are incorrect or unable to provide a grading, and it will be difficult to determine this value from study to study. There is also a high variability in how results are reported by various studies. For example, some studies may report a diagnosis of the ophthalmic condition at any severity, at a specific severity, or at referable disease, of which the definition may also vary [26]. Due to the novelty of this topic, we anticipate only a small number of randomized control trials which are typically believed to provide the highest level of evidence [27].

Overall, this systematic review and meta-analysis will provide novel insights into the diagnostic accuracy of AI in new domains of ophthalmology that have not been previously studied. The results from our review may help to either support the use of AI in specific applications in ophthalmology or point out areas of weakness in which AI lacks the reliability to be used in lieu of human graders. This protocol also documents the use of an AI-based software to assist article screening, which may serve as a reference for future large systematic reviews to make screening more accessible.

## Supplementary Information

**Additional file 1. **Search Strategy.

## Data Availability

Not applicable

## Abbreviations

AI: Artificial intelligence
QUADAS-2: Quality Assessment of Diagnostic Accuracy Studies
sROC: Summary receiver operating characteristic
AS-OCT: Anterior segment optical coherence tomography
PRISMA-DTA: Preferred Reporting Items for Systematic Reviews and Meta-Analyses for Diagnostic Test Accuracy
AUC: Area under the curve
NPV: Negative predictive value
PPV: Positive predictive value
