# Supplementary material for: Protocol for a systematic review and meta-analysis of the diagnostic accuracy of artificial intelligence for grading of ophthalmology imaging modalities
Source: Diagn Progn Res. 2022 Jul 14;6:15. doi: 10.1186/s41512-022-00127-9 (PMC9281030; doi:10.1186/s41512-022-00127-9)
Supplement: Supplementary file 1 — Additional file 1. Search Strategy. [file 41512_2022_127_MOESM1_ESM.docx]

OVID MEDLINE

Database(s): **Ovid MEDLINE: Epub Ahead of Print, In-Process & Other Non-Indexed Citations, Ovid MEDLINE® Daily and Ovid MEDLINE®**1946-Present
Search Strategy:

| **#** | **Searches** | **Results** |
| --- | --- | --- |
| 1 | artificial intelligence/ | 28849 |
| 2 | (artificial adj2 intelligence).mp. | 40229 |
| 3 | machine learning/ | 20631 |
| 4 | (machine adj2 learning).mp. | 60632 |
| 5 | deep learning/ | 8587 |
| 6 | (deep adj2 learn*).mp. | 27083 |
| 7 | supervised machine learning/ | 1098 |
| 8 | (supervised adj2 learn*).mp. | 6229 |
| 9 | unsupervised machine learning/ | 602 |
| 10 | (unsupervised adj2 learn*).mp. | 3224 |
| 11 | support vector machine/ | 8545 |
| 12 | (support adj2 vector adj2 machine*).mp. | 21684 |
| 13 | neural networks, computer/ | 35217 |
| 14 | "neural network*".mp. | 79070 |
| 15 | pattern recognition, automated/ | 26082 |
| 16 | (automated adj2 pattern* adj2 recognition).mp. | 26146 |
| 17 | diagnosis, computer-assisted/ or image interpretation, computer-assisted/ | 70441 |
| 18 | (("computer-assisted" or "computer assisted") adj2 (diagnos* or "image interpret*")).mp. | 85865 |
| 19 | "computational intelligence".mp. | 366 |
| 20 | bayes theorem/ | 40532 |
| 21 | (bayes adj2 theorem*).mp. | 41085 |
| 22 | (naive adj2 bayes).mp. | 2194 |
| 23 | Decision Trees/ | 11788 |
| 24 | (decision adj2 tree*).mp. | 19761 |
| 25 | (random adj2 forest*).mp. | 14051 |
| 26 | (boosted adj2 tree*).mp. | 928 |
| 27 | (reinforcement adj2 learn*).mp. | 4625 |
| 28 | or/1-27 [****AI Terms****] | 315939 |
| 29 | Vision Screening/ | 2356 |
| 30 | tele?ophthalmolog*.mp. | 306 |
| 31 | tele?glaucoma*.mp. | 23 |
| 32 | tele?retina*.mp. | 54 |
| 33 | or/29-32 [****Teleophthalmology Terms****] | 2716 |
| 34 | Tomography, Optical Coherence/ | 40985 |
| 35 | "Optical coherence tomography".mp. | 43443 |
| 36 | OCT.mp. | 42420 |
| 37 | Corneal Topography/ | 6879 |
| 38 | Visual Fields/ | 31718 |
| 39 | "Visual Field*".mp. | 49602 |
| 40 | (slit adj2 lamp adj2 (photo* or imag*)).mp. | 677 |
| 41 | ((fundus or fundal) adj2 (photo* or imag*)).mp. | 9601 |
| 42 | "Ultrasound* biomicroscopy".mp. | 1889 |
| 43 | "Fluorescein angiogra*".mp. | 32073 |
| 44 | "Indocyanine green angiogra*".mp. | 2895 |
| 45 | (Ocular adj (ultrasonography or ultrasound)).mp. | 460 |
| 46 | Microperimetry.mp. | 1237 |
| 47 | Optos.mp. | 234 |
| 48 | "Specular microscopy".mp. | 1473 |
| 49 | "Optic nerve head topography".mp. | 94 |
| 50 | (Ultra wide field adj2 (imag* or photo*)).mp. | 141 |
| 51 | (Wide field adj2 angiogra*).mp. | 154 |
| 52 | or/34-51 [***imaging terms***] | 147988 |
| 53 | corneal diseases/ | 13540 |
| 54 | corneal opacity/ | 3917 |
| 55 | keratitis/ | 8631 |
| 56 | keratitis.mp. | 20484 |
| 57 | keratoconus/ | 6035 |
| 58 | keratoconus.mp. | 8166 |
| 59 | lens diseases/ | 889 |
| 60 | cataract/ | 30252 |
| 61 | cataract*.mp. | 73426 |
| 62 | capsule opacification/ | 491 |
| 63 | "capsule opacification".mp. | 1715 |
| 64 | retinal diseases/ | 22213 |
| 65 | diabetic retinopathy/ | 26940 |
| 66 | epiretinal membrane/ | 2480 |
| 67 | "epiretinal membrane*".mp. | 4582 |
| 68 | hypertensive retinopathy/ | 215 |
| 69 | retinal arterial macroaneurysm/ | 32 |
| 70 | retinal artery occlusion/ | 2372 |
| 71 | retinal degeneration/ | 10454 |
| 72 | macular degeneration/ | 16402 |
| 73 | "macular degenerat*".mp. | 28089 |
| 74 | retinal drusen/ | 1154 |
| 75 | retinal detachment/ | 20144 |
| 76 | retinal hemorrhage/ | 5455 |
| 77 | retinal neovascularization/ | 3227 |
| 78 | retinal vasculitis/ | 776 |
| 79 | retinal vein occlusion/ | 4581 |
| 80 | retinitis/ | 3530 |
| 81 | retinitis.mp. | 18425 |
| 82 | chorioretinitis/ | 2511 |
| 83 | chorioretinitis.mp. | 3315 |
| 84 | "retinopathy of prematurity"/ | 6475 |
| 85 | vitreoretinopathy, proliferative/ | 1677 |
| 86 | scleral diseases/ | 969 |
| 87 | uveal diseases/ | 1786 |
| 88 | choroid diseases/ | 3098 |
| 89 | iris diseases/ | 2561 |
| 90 | (iris adj2 diseas*).mp. | 2616 |
| 91 | iridocyclitis.mp. | 1740 |
| 92 | iritis.mp. | 1977 |
| 93 | (iris adj (neoplasm* or tumor* or tumour* or malign* or cancer*)).mp. | 850 |
| 94 | uveitis/ | 12521 |
| 95 | panuveitis/ | 592 |
| 96 | uveitis, anterior/ | 3547 |
| 97 | uveitis, posterior/ | 752 |
| 98 | uveitis, intermediate/ | 461 |
| 99 | vitreous detachment/ | 851 |
| 100 | Strabismus/ | 13617 |
| 101 | Strabismus.mp. | 19195 |
| 102 | eyelid diseases/ | 7210 |
| 103 | blepharitis/ | 1384 |
| 104 | blepharitis.mp. | 2182 |
| 105 | chalazion/ | 273 |
| 106 | chalazion.mp. | 590 |
| 107 | ectropion/ | 1207 |
| 108 | ectropion.mp. | 2384 |
| 109 | entropion/ | 967 |
| 110 | entropion.mp. | 1427 |
| 111 | hordeolum/ | 129 |
| 112 | hordeolum.mp. | 190 |
| 113 | eye neoplasms/ | 12273 |
| 114 | conjunctival neoplasms/ | 2810 |
| 115 | eyelid neoplasms/ | 4746 |
| 116 | orbital neoplasms/ | 9395 |
| 117 | retinal neoplasms/ | 3892 |
| 118 | retinoblastoma/ | 7771 |
| 119 | retinoblastoma*.mp. | 23587 |
| 120 | uveal neoplasms/ | 4630 |
| 121 | choroid neoplasms/ | 5501 |
| 122 | iris neoplasms/ | 762 |
| 123 | retinopath*.mp. | 59980 |
| 124 | proliferative vitreoretinopath*.mp. | 2798 |
| 125 | uveitis.mp. | 24943 |
| 126 | panuveitis.mp. | 1800 |
| 127 | (conjunctival adj (neoplasm* or tumor* or tumour* or malignan* or cancer*)).mp. | 2972 |
| 128 | (orbital adj (neoplasm* or tumor* or tumour* or malignan* or cancer*)).mp. | 10080 |
| 129 | maculopath*.mp. | 5094 |
| 130 | (macula* adj2 (edema* or oedema*)).mp. | 14749 |
| 131 | or/53-130 [***eye pathology***] | 365451 |
| 132 | eyelids/ | 14914 |
| 133 | eyelid*.mp. | 37481 |
| 134 | eyelashes/ | 1421 |
| 135 | eyelash*.mp. | 2712 |
| 136 | uvea/ | 1908 |
| 137 | uvea*.mp. | 11974 |
| 138 | anterior eye segment/ | 4159 |
| 139 | anterior chamber/ | 10181 |
| 140 | aqueous humor/ | 11492 |
| 141 | endothelium, corneal/ | 6609 |
| 142 | epithelium, corneal/ | 6192 |
| 143 | limbus corneae/ | 2302 |
| 144 | corneal stroma/ | 6136 |
| 145 | cornea/ | 48995 |
| 146 | cornea*.mp. | 122921 |
| 147 | ciliary body/ | 8361 |
| 148 | (ciliary adj2 (body or bodies)).mp. | 11469 |
| 149 | conjunctiva/ | 16215 |
| 150 | bowman membrane/ | 156 |
| 151 | descemet membrane/ | 2239 |
| 152 | iris/ | 13599 |
| 153 | pupil/ | 9980 |
| 154 | lens, crystalline/ | 23526 |
| 155 | lens capsule, crystalline/ | 4152 |
| 156 | "posterior capsule of the lens"/ | 293 |
| 157 | "anterior capsule of the lens"/ | 227 |
| 158 | lens cortex, crystalline/ | 526 |
| 159 | lens nucleus, crystalline/ | 774 |
| 160 | trabecular meshwork/ | 4377 |
| 161 | axial length, eye/ | 1670 |
| 162 | meibomian glands/ | 1674 |
| 163 | meibomian.mp. | 2699 |
| 164 | lacrimal apparatus/ | 8183 |
| 165 | lacrimal.mp. | 17971 |
| 166 | nasolacrimal duct/ | 2567 |
| 167 | "pigment epithelium of eye"/ | 9447 |
| 168 | posterior eye segment/ | 428 |
| 169 | vitreous body/ | 19684 |
| 170 | vitreous.mp. | 37163 |
| 171 | blood-retinal barrier/ | 1472 |
| 172 | fundus oculi/ | 18188 |
| 173 | macula lutea/ | 10287 |
| 174 | macula*.mp. | 75687 |
| 175 | fovea centralis/ | 4982 |
| 176 | fovea.mp. | 10819 |
| 177 | optic disk/ | 11275 |
| 178 | ("optic disc" or "optic discs" or "optic disk" or "optic disks").mp. | 18197 |
| 179 | amacrine cells/ | 1040 |
| 180 | rod cell outer segment/ | 2791 |
| 181 | retinal pigment epithelium/ | 6884 |
| 182 | retinal neurons/ | 1060 |
| 183 | photoreceptor cells/ | 12661 |
| 184 | photoreceptor cells, invertebrate/ | 2776 |
| 185 | photoreceptor cells, vertebrate/ | 4025 |
| 186 | photoreceptor connecting cilium/ | 30 |
| 187 | retinal cone photoreceptor cells/ | 5004 |
| 188 | retinal photoreceptor cell inner segment/ | 267 |
| 189 | retinal photoreceptor cell outer segment/ | 571 |
| 190 | retinal rod photoreceptor cells/ | 3530 |
| 191 | photoreceptor.mp. | 38295 |
| 192 | retinal bipolar cells/ | 801 |
| 193 | retinal ganglion cells/ | 16086 |
| 194 | retinal horizontal cells/ | 275 |
| 195 | retina/ | 77815 |
| 196 | retina*.mp. | 255460 |
| 197 | sclera/ | 11427 |
| 198 | Sclera*.mp. | 26961 |
| 199 | tenon capsule/ | 297 |
| 200 | choroid/ | 13860 |
| 201 | Choroid.mp. | 36490 |
| 202 | bruch membrane/ | 946 |
| 203 | "bruch membrane*".mp. | 1166 |
| 204 | or/132-203 [***eye structures***] | 579176 |
| 205 | eye/ | 40282 |
| 206 | (eye or eyes).mp. | 493487 |
| 207 | ophthalmology/ | 23925 |
| 208 | ophthalmolog*.mp. | 89924 |
| 209 | or/205-208 [***general eye terms***] | 540110 |
| 210 | 33 or 52 or 131 or 204 or 209 | 1002033 |
| 211 | 28 and 210 [****base set****] | 11075 |
| 212 | limit 211 to yr="2000 -Current" | 10089 |

EMBASE

Database(s): **Embase Classic+Embase**1947 to 2021 December 17
Search Strategy:

| **#** | **Searches** | **Results** |
| --- | --- | --- |
| 1 | artificial intelligence/ | 36816 |
| 2 | (artificial adj2 intelligence).mp. | 41740 |
| 3 | machine learning/ | 49692 |
| 4 | (machine adj2 learning).mp. | 80143 |
| 5 | deep learning/ | 20350 |
| 6 | (deep adj2 learn*).mp. | 31167 |
| 7 | supervised machine learning/ | 2455 |
| 8 | (supervised adj2 learn*).mp. | 7652 |
| 9 | unsupervised machine learning/ | 1348 |
| 10 | (unsupervised adj2 learn*).mp. | 3982 |
| 11 | support vector machine/ | 28033 |
| 12 | (support adj2 vector adj2 machine*).mp. | 33388 |
| 13 | artificial neural network/ | 41349 |
| 14 | "neural network*".mp. | 97644 |
| 15 | automated pattern recognition/ | 17216 |
| 16 | (automated adj2 pattern* adj2 recognition).mp. | 17292 |
| 17 | computer assisted diagnosis/ | 42350 |
| 18 | (("computer-assisted" or "computer assisted") adj2 (diagnos* or "image interpret*")).mp. | 43060 |
| 19 | "computational intelligence".mp. | 495 |
| 20 | bayes theorem/ | 37808 |
| 21 | (bayes adj2 theorem*).mp. | 38476 |
| 22 | (naive adj2 bayes).mp. | 2754 |
| 23 | Decision Tree/ | 16262 |
| 24 | (decision adj2 tree*).mp. | 21991 |
| 25 | (random adj2 forest*).mp. | 19876 |
| 26 | (boosted adj2 tree*).mp. | 1115 |
| 27 | (reinforcement adj2 learn*).mp. | 5404 |
| 28 | or/1-27 [****AI Terms****] | 313783 |
| 29 | Vision test/ | 10918 |
| 30 | tele?ophthalmolog*.mp. | 402 |
| 31 | tele?glaucoma*.mp. | 32 |
| 32 | tele?retina*.mp. | 100 |
| 33 | or/29-32 [****Teleophthalmology Terms****] | 11433 |
| 34 | optical coherence tomography/ | 60329 |
| 35 | "Optical coherence tomography".mp. | 81022 |
| 36 | OCT.mp. | 73169 |
| 37 | keratometry/ | 9359 |
| 38 | Visual Field/ | 39671 |
| 39 | "Visual Field*".mp. | 64362 |
| 40 | (slit adj2 lamp adj2 (photo* or imag*)).mp. | 935 |
| 41 | ((fundus or fundal) adj2 (photo* or imag*)).mp. | 14052 |
| 42 | "Ultrasound* biomicroscopy".mp. | 2300 |
| 43 | "Fluorescein angiogra*".mp. | 19067 |
| 44 | "Indocyanine green angiogra*".mp. | 6224 |
| 45 | (ocular adj (ultrasonography or ultrasound)).mp. | 589 |
| 46 | Microperimetry.mp. | 1835 |
| 47 | Optos.mp. | 1003 |
| 48 | "Specular microscopy".mp. | 2531 |
| 49 | "Optic nerve head topography".mp. | 117 |
| 50 | (Ultra wide field adj2 (imag* or photo*)).mp. | 220 |
| 51 | (Wide field adj2 angiogra*).mp. | 267 |
| 52 | or/34-51 [***imaging terms***] | 203390 |
| 53 | cornea disease/ | 11658 |
| 54 | cornea opacity/ | 8000 |
| 55 | keratitis/ | 17634 |
| 56 | keratitis.mp. | 30087 |
| 57 | keratoconus/ | 10208 |
| 58 | keratoconus.mp. | 11024 |
| 59 | lens disease/ | 1657 |
| 60 | cataract/ | 61777 |
| 61 | cataract*.mp. | 112427 |
| 62 | capsule opacification/ | 773 |
| 63 | "capsule opacification".mp. | 2434 |
| 64 | retina disease/ | 15710 |
| 65 | diabetic retinopathy/ | 47513 |
| 66 | epiretinal membrane/ | 6204 |
| 67 | "epiretinal membrane*".mp. | 7188 |
| 68 | hypertension retinopathy/ | 1719 |
| 69 | retina artery occlusion/ | 2719 |
| 70 | retina degeneration/ | 13805 |
| 71 | macular degeneration/ | 3930 |
| 72 | "macular degenerat*".mp. | 37008 |
| 73 | drusen/ | 3823 |
| 74 | retina detachment/ | 37355 |
| 75 | retina hemorrhage/ | 9590 |
| 76 | retina neovascularization/ | 6547 |
| 77 | retina vasculitis/ | 2552 |
| 78 | retina vein occlusion/ | 5491 |
| 79 | retinitis/ | 8208 |
| 80 | retinitis.mp. | 27558 |
| 81 | chorioretinitis/ | 4751 |
| 82 | chorioretinitis.mp. | 5383 |
| 83 | retrolental fibroplasia/ | 11662 |
| 84 | vitreoretinopathy/ | 5416 |
| 85 | sclera disease/ | 1603 |
| 86 | uvea disease/ | 1137 |
| 87 | choroid disease/ | 3413 |
| 88 | iris disease/ | 3028 |
| 89 | (iris adj2 diseas*).mp. | 3159 |
| 90 | iridocyclitis.mp. | 9471 |
| 91 | iritis.mp. | 4202 |
| 92 | (iris adj (neoplasm* or tumor* or tumour* or malign* or cancer*)).mp. | 1035 |
| 93 | uveitis/ | 30793 |
| 94 | intermediate uveitis/ | 1311 |
| 95 | vitreous body detachment/ | 2752 |
| 96 | Strabismus/ | 21038 |
| 97 | Strabismus.mp. | 34059 |
| 98 | eyelid disease/ | 6597 |
| 99 | blepharitis/ | 3872 |
| 100 | blepharitis.mp. | 4314 |
| 101 | chalazion/ | 1030 |
| 102 | chalazion.mp. | 1204 |
| 103 | ectropion/ | 3425 |
| 104 | ectropion.mp. | 4197 |
| 105 | entropion/ | 1846 |
| 106 | entropion.mp. | 2123 |
| 107 | hordeolum/ | 577 |
| 108 | hordeolum.mp. | 607 |
| 109 | eye tumor/ | 8363 |
| 110 | conjunctiva tumor/ | 2560 |
| 111 | eyelid tumor/ | 3336 |
| 112 | orbit tumor/ | 8569 |
| 113 | retina tumor/ | 1662 |
| 114 | retinoblastoma/ | 14906 |
| 115 | retinoblastoma*.mp. | 33903 |
| 116 | uvea tumor/ | 1089 |
| 117 | choroid tumor/ | 3299 |
| 118 | iris tumor/ | 910 |
| 119 | retinopath*.mp. | 98906 |
| 120 | proliferative vitreoretinopath*.mp. | 3438 |
| 121 | uveitis.mp. | 41062 |
| 122 | panuveitis.mp. | 2291 |
| 123 | (conjunctival adj (neoplasm* or tumor* or tumour* or malignan* or cancer*)).mp. | 802 |
| 124 | (orbital adj (neoplasm* or tumor* or tumour* or malignan* or cancer*)).mp. | 3443 |
| 125 | maculopath*.mp. | 10439 |
| 126 | (macula* adj2 (edema* or oedema*)).mp. | 25953 |
| 127 | or/53-126 [***eye pathology***] | 509267 |
| 128 | eyelid/ | 16107 |
| 129 | eyelid*.mp. | 61852 |
| 130 | eyelash/ | 2998 |
| 131 | eyelash*.mp. | 4193 |
| 132 | uvea/ | 2840 |
| 133 | uvea*.mp. | 15515 |
| 134 | anterior eye segment/ | 5975 |
| 135 | anterior eye chamber/ | 19660 |
| 136 | aqueous humor/ | 14459 |
| 137 | cornea endothelium/ | 8727 |
| 138 | cornea epithelium/ | 13680 |
| 139 | cornea limbus/ | 5005 |
| 140 | cornea stroma/ | 6017 |
| 141 | cornea/ | 45922 |
| 142 | cornea*.mp. | 168961 |
| 143 | ciliary body/ | 9212 |
| 144 | (ciliary adj2 (body or bodies)).mp. | 14148 |
| 145 | conjunctiva/ | 20708 |
| 146 | bowman membrane/ | 1293 |
| 147 | descemet membrane/ | 3650 |
| 148 | iris/ | 17491 |
| 149 | pupil/ | 12239 |
| 150 | lens/ | 30374 |
| 151 | lens capsule/ | 4176 |
| 152 | posterior lens capsule/ | 425 |
| 153 | anterior lens capsule/ | 628 |
| 154 | lens cortex/ | 155 |
| 155 | lens nucleus/ | 860 |
| 156 | trabecular meshwork/ | 6565 |
| 157 | eye axial length/ | 4294 |
| 158 | meibomian gland/ | 2389 |
| 159 | meibomian.mp. | 4136 |
| 160 | lacrimal apparatus/ | 3890 |
| 161 | lacrimal.mp. | 36546 |
| 162 | nasolacrimal duct/ | 3867 |
| 163 | posterior eye segment/ | 719 |
| 164 | vitreous body/ | 22707 |
| 165 | vitreous.mp. | 49280 |
| 166 | blood-retinal barrier/ | 3136 |
| 167 | eye fundus/ | 19288 |
| 168 | retina macula lutea/ | 13985 |
| 169 | macula*.mp. | 113616 |
| 170 | retina fovea/ | 13801 |
| 171 | fovea.mp. | 18963 |
| 172 | optic disk/ | 15606 |
| 173 | (optic disc or optic discs or optic disk or optic disks).mp. | 24583 |
| 174 | retina amacrine cell/ | 4051 |
| 175 | rod cell outer segment/ | 350 |
| 176 | retinal pigment epithelium/ | 8518 |
| 177 | retina nerve cell/ | 5600 |
| 178 | photoreceptor cell/ | 8569 |
| 179 | invertebrate photoreceptor cell/ | 134 |
| 180 | vertebrate photoreceptor cell/ | 562 |
| 181 | photoreceptor connecting cilium/ | 185 |
| 182 | retina cone/ | 9354 |
| 183 | photoreceptor inner segment/ | 879 |
| 184 | photoreceptor outer segment/ | 2063 |
| 185 | retina rod/ | 7650 |
| 186 | photoreceptor.mp. | 45835 |
| 187 | retina bipolar ganglion cell/ | 3342 |
| 188 | retina ganglion cell/ | 20773 |
| 189 | retina horizontal nerve cell/ | 1348 |
| 190 | retina/ | 72096 |
| 191 | retina*.mp. | 365928 |
| 192 | sclera/ | 14631 |
| 193 | Sclera*.mp. | 34579 |
| 194 | tenon capsule/ | 542 |
| 195 | choroid/ | 12946 |
| 196 | Choroid.mp. | 51582 |
| 197 | bruch membrane/ | 3395 |
| 198 | "bruch membrane*".mp. | 3545 |
| 199 | or/128-198 [***eye structures***] | 788490 |
| 200 | eye/ | 92054 |
| 201 | (eye or eyes).mp. | 708065 |
| 202 | ophthalmology/ | 33952 |
| 203 | ophthalmolog*.mp. | 117917 |
| 204 | or/200-203 [***general eye terms***] | 762295 |
| 205 | 33 or 52 or 127 or 199 or 204 | 1408032 |
| 206 | 28 and 205 [****base set****] | 13426 |
| 207 | limit 206 to yr="2000 -Current" | 12592 |

COCHRANE

Search Name: Cochrane Dec 20

Date Run: 21/12/2021 01:30:57

Comment: Ophthal and AI

ID Search Hits

#1 MeSH descriptor: [Artificial Intelligence] explode all trees 1249

#2 (Artificial near/2 intelligence) 975

#3 MeSH descriptor: [Machine Learning] explode all trees 168

#4 (machine near/2 learning) 1690

#5 MeSH descriptor: [Deep Learning] explode all trees 33

#6 (deep near/2 learn*) 681

#7 MeSH descriptor: [Supervised Machine Learning] explode all trees 21

#8 (supervised near/2 learn*) 124

#9 MeSH descriptor: [Unsupervised Machine Learning] explode all trees 0

#10 (unsupervised near/2 learn*) 44

#11 MeSH descriptor: [Support Vector Machine] explode all trees 19

#12 (support near/2 vector near/2 machine*) 380

#13 MeSH descriptor: [Neural Networks, Computer] explode all trees 148

#14 "neural network*" 1063

#15 MeSH descriptor: [Pattern Recognition, Automated] explode all trees 193

#16 (automated NEAR/2 pattern* NEAR/2 recognition) 199

#17 MeSH descriptor: [Diagnosis, Computer-Assisted] explode all trees 1929

#18 MeSH descriptor: [Image Interpretation, Computer-Assisted] explode all trees 7929

#19 (("computer-assisted" or "computer assisted") near/2 (diagnos* or "image interpret*")) 1093

#20 "computational intelligence" 9

#21 MeSH descriptor: [Bayes Theorem] explode all trees 521

#22 (bayes near/2 theorem*) 944

#23 (naive near/2 bayes) 32

#24 MeSH descriptor: [Decision Trees] explode all trees 165

#25 (decision near/2 tree*) 836

#26 (random near/2 forest*) 547

#27 (boosted near/2 tree*) 17

#28 (reinforcement near/2 learn*) 198

#29 {OR #1-#28} 15241

#30 MeSH descriptor: [Vision Screening] explode all trees 97

#31 tele?ophthalmolog* 18

#32 tele?glaucoma* 5

#33 tele?retina* 4

#34 {OR #30-#33} 121

#35 MeSH descriptor: [Tomography, Optical Coherence] explode all trees 1522

#36 "Optical coherence tomography" 3864

#37 OCT 19889

#38 MeSH descriptor: [Corneal Topography] explode all trees 403

#39 MeSH descriptor: [Visual Fields] explode all trees 924

#40 "Visual Field*" 3091

#41 (slit NEAR/2 lamp NEAR/2 (photo* or imag*)) 90

#42 ((fundus or fundal) NEAR/2 (photo* or imag*)) 1149

#43 "Ultrasound* biomicroscopy" 77

#44 "Fluorescein angiogra*" 0

#45 "Indocyanine green angiogra*" 0

#46 (ocular NEXT (ultrasonography or ultrasound)) 39

#47 Microperimetry 234

#48 Optos 140

#49 "Specular microscopy" 260

#50 "Optic nerve head topography" 6

#51 (Ultra NEXT wide NEXT field NEAR/2 (imag* or photo*)) 5

#52 (wide NEXT field NEAR/2 angiogra*) 17

#53 {OR #35-#52} 26319

#54 MeSH descriptor: [Corneal Diseases] explode all trees 2165

#55 MeSH descriptor: [Corneal Opacity] explode all trees 71

#56 MeSH descriptor: [Keratitis] explode all trees 1035

#57 keratitis 1255

#58 MeSH descriptor: [Keratoconus] explode all trees 253

#59 keratoconus 635

#60 MeSH descriptor: [Lens Diseases] explode all trees 1633

#61 MeSH descriptor: [Cataract] explode all trees 1541

#62 cataract* 8971

#63 MeSH descriptor: [Capsule Opacification] explode all trees 110

#64 "capsule opacification" 371

#65 MeSH descriptor: [Retinal Diseases] explode all trees 5344

#66 MeSH descriptor: [Diabetic Retinopathy] explode all trees 1507

#67 MeSH descriptor: [Epiretinal Membrane] explode all trees 120

#68 "epiretinal membrane*" 387

#69 MeSH descriptor: [Hypertensive Retinopathy] explode all trees 2

#70 MeSH descriptor: [Retinal Arterial Macroaneurysm] explode all trees 0

#71 MeSH descriptor: [Retinal Artery Occlusion] explode all trees 31

#72 MeSH descriptor: [Retinal Degeneration] explode all trees 2827

#73 MeSH descriptor: [Macular Degeneration] explode all trees 2689

#74 "macular degenerat*" 1

#75 MeSH descriptor: [Retinal Drusen] explode all trees 68

#76 MeSH descriptor: [Retinal Detachment] explode all trees 352

#77 MeSH descriptor: [Retinal Hemorrhage] explode all trees 65

#78 MeSH descriptor: [Retinal Neovascularization] explode all trees 83

#79 MeSH descriptor: [Retinal Vasculitis] explode all trees 12

#80 MeSH descriptor: [Retinal Vein Occlusion] explode all trees 378

#81 MeSH descriptor: [Retinitis] explode all trees 187

#82 retinitis 534

#83 MeSH descriptor: [Chorioretinitis] explode all trees 22

#84 chorioretinitis 73

#85 MeSH descriptor: [Retinopathy of Prematurity] explode all trees 406

#86 MeSH descriptor: [Vitreoretinopathy, Proliferative] explode all trees 64

#87 MeSH descriptor: [Scleral Diseases] explode all trees 16

#88 MeSH descriptor: [Uveal Diseases] explode all trees 1417

#89 MeSH descriptor: [Choroid Diseases] explode all trees 590

#90 MeSH descriptor: [Iris Diseases] explode all trees 213

#91 (iris adj2 diseas*) 77

#92 iridocyclitis 169

#93 iritis 170

#94 (iris NEXT (neoplasm* or tumor* or tumour* or malign* or cancer*)) 1

#95 MeSH descriptor: [Uveitis] explode all trees 641

#96 MeSH descriptor: [Panuveitis] explode all trees 411

#97 MeSH descriptor: [Uveitis, Anterior] explode all trees 327

#98 MeSH descriptor: [Uveitis, Posterior] explode all trees 91

#99 MeSH descriptor: [Uveitis, Intermediate] explode all trees 47

#100 MeSH descriptor: [Vitreous Detachment] explode all trees 39

#101 MeSH descriptor: [Strabismus] explode all trees 558

#102 strabismus 1440

#103 MeSH descriptor: [Eyelid Diseases] explode all trees 514

#104 MeSH descriptor: [Blepharitis] explode all trees 129

#105 blepharitis 363

#106 MeSH descriptor: [Chalazion] explode all trees 23

#107 chalazion 59

#108 MeSH descriptor: [Ectropion] explode all trees 12

#109 ectropion 74

#110 MeSH descriptor: [Entropion] explode all trees 15

#111 entropion 70

#112 MeSH descriptor: [Hordeolum] explode all trees 7

#113 hordeolum 62

#114 MeSH descriptor: [Eye Neoplasms] explode all trees 193

#115 MeSH descriptor: [Conjunctival Neoplasms] explode all trees 9

#116 MeSH descriptor: [Eyelid Neoplasms] explode all trees 7

#117 MeSH descriptor: [Orbital Neoplasms] explode all trees 9

#118 MeSH descriptor: [Retinal Neoplasms] explode all trees 26

#119 MeSH descriptor: [Retinoblastoma] explode all trees 25

#120 retinoblastoma* 173

#121 MeSH descriptor: [Uveal Neoplasms] explode all trees 120

#122 MeSH descriptor: [Choroid Neoplasms] explode all trees 50

#123 MeSH descriptor: [Iris Neoplasms] explode all trees 0

#124 retinopath* 6708

#125 "proliferative vitreoretinopath*" 0

#126 uveitis 1476

#127 panuveitis 175

#128 (conjunctival NEXT (neoplasm* or tumor* or tumour* or malignan* or cancer*)) 12

#129 (orbital NEXT (neoplasm* or tumor* or tumour* or malignan* or cancer*)) 15

#130 maculopath* 471

#131 (macula* near/2 (edema* or oedema*)) 3673

#132 {OR #54-#131} 25949

#133 MeSH descriptor: [Eyelids] explode all trees 1058

#134 eyelid* 2234

#135 MeSH descriptor: [Eyelashes] explode all trees 57

#136 eyelash* 513

#137 MeSH descriptor: [Uvea] explode all trees 1191

#138 uvea* 294

#139 MeSH descriptor: [Anterior Eye Segment] explode all trees 4349

#140 MeSH descriptor: [Anterior Chamber] explode all trees 1058

#141 MeSH descriptor: [Aqueous Humor] explode all trees 378

#142 MeSH descriptor: [Endothelium, Corneal] explode all trees 389

#143 MeSH descriptor: [Limbus Corneae] explode all trees 53

#144 MeSH descriptor: [Epithelium, Corneal] explode all trees 182

#145 MeSH descriptor: [Corneal Stroma] explode all trees 239

#146 MeSH descriptor: [Cornea] explode all trees 1947

#147 cornea* 9724

#148 MeSH descriptor: [Ciliary Body] explode all trees 82

#149 (ciliary near/2 (body or bodies)) 225

#150 MeSH descriptor: [Conjunctiva] explode all trees 704

#151 MeSH descriptor: [Bowman Membrane] explode all trees 7

#152 MeSH descriptor: [Descemet Membrane] explode all trees 26

#153 MeSH descriptor: [Iris] explode all trees 838

#154 MeSH descriptor: [Pupil] explode all trees 690

#155 MeSH descriptor: [Lens, Crystalline] explode all trees 425

#156 MeSH descriptor: [Lens Capsule, Crystalline] explode all trees 280

#157 MeSH descriptor: [Posterior Capsule of the Lens] explode all trees 36

#158 MeSH descriptor: [Anterior Capsule of the Lens] explode all trees 12

#159 MeSH descriptor: [Lens Cortex, Crystalline] explode all trees 12

#160 MeSH descriptor: [Lens Nucleus, Crystalline] explode all trees 30

#161 MeSH descriptor: [Trabecular Meshwork] explode all trees 103

#162 MeSH descriptor: [Axial Length, Eye] explode all trees 64

#163 MeSH descriptor: [Meibomian Glands] explode all trees 88

#164 meibomian 501

#165 MeSH descriptor: [Lacrimal Apparatus] explode all trees 150

#166 lacrimal 962

#167 MeSH descriptor: [Nasolacrimal Duct] explode all trees 72

#168 MeSH descriptor: [Pigment Epithelium of Eye] explode all trees 78

#169 MeSH descriptor: [Posterior Eye Segment] explode all trees 451

#170 MeSH descriptor: [Vitreous Body] explode all trees 438

#171 vitreous 2002

#172 MeSH descriptor: [Blood-Retinal Barrier] explode all trees 33

#173 MeSH descriptor: [Fundus Oculi] explode all trees 378

#174 MeSH descriptor: [Macula Lutea] explode all trees 491

#175 macula* 8998

#176 MeSH descriptor: [Fovea Centralis] explode all trees 144

#177 fovea 894

#178 MeSH descriptor: [Optic Disk] explode all trees 244

#179 ("optic disc" or "optic discs" or "optic disk" or "optic disks") 811

#180 MeSH descriptor: [Amacrine Cells] explode all trees 0

#181 MeSH descriptor: [Rod Cell Outer Segment] explode all trees 1

#182 MeSH descriptor: [Retinal Pigment Epithelium] explode all trees 57

#183 MeSH descriptor: [Retinal Neurons] explode all trees 181

#184 MeSH descriptor: [Photoreceptor Cells] explode all trees 64

#185 MeSH descriptor: [Photoreceptor Cells, Invertebrate] explode all trees 0

#186 MeSH descriptor: [Photoreceptor Cells, Vertebrate] explode all trees 53

#187 MeSH descriptor: [Photoreceptor Connecting Cilium] explode all trees 0

#188 MeSH descriptor: [Retinal Cone Photoreceptor Cells] explode all trees 22

#189 MeSH descriptor: [Retinal Photoreceptor Cell Inner Segment] explode all trees 6

#190 MeSH descriptor: [Retinal Photoreceptor Cell Outer Segment] explode all trees 9

#191 MeSH descriptor: [Retinal Rod Photoreceptor Cells] explode all trees 15

#192 photoreceptor 245

#193 MeSH descriptor: [Retinal Bipolar Cells] explode all trees 1

#194 MeSH descriptor: [Retinal Ganglion Cells] explode all trees 116

#195 MeSH descriptor: [Retinal Horizontal Cells] explode all trees 0

#196 MeSH descriptor: [Retina] explode all trees 1752

#197 retina* 11225

#198 MeSH descriptor: [Sclera] explode all trees 233

#199 sclera* 1381

#200 MeSH descriptor: [Tenon Capsule] explode all trees 20

#201 MeSH descriptor: [Choroiditis] explode all trees 50

#202 choroid 771

#203 MeSH descriptor: [Bruch Membrane] explode all trees 3

#204 "bruch membrane*" 63

#205 {OR #133-#204} 30497

#206 MeSH descriptor: [Eye] explode all trees 7415

#207 (eye or eyes) 52591

#208 MeSH descriptor: [Ophthalmology] explode all trees 138

#209 ophthalmolog* 23274

#210 {OR #206-#209} 56766

#211 #34 OR #53 OR #132 OR #205 OR #210 84187

#212 #29 AND #211 639

#213 #212 with Cochrane Library publication date Between Jan 2000 and Dec 2021 588
